# Longitudinal, Lateral and Transverse Axes of Forearm Muscles Influence the Crosstalk in the Mechanomyographic Signals during Isometric Wrist Postures

**DOI:** 10.1371/journal.pone.0104280

**Published:** 2014-08-04

**Authors:** Md. Anamul Islam, Kenneth Sundaraj, R. Badlishah Ahmad, Sebastian Sundaraj, Nizam Uddin Ahamed, Md. Asraf Ali

**Affiliations:** 1 AI-Rehab Research Group, Universiti Malaysia Perlis, Arau, Perlis, Malaysia; 2 Malaysian Ministry of Health, Klang, Selangor, Malaysia; University of Rome Foro Italico, Italy

## Abstract

**Problem Statement:**

In mechanomyography (MMG), crosstalk refers to the contamination of the signal from the muscle of interest by the signal from another muscle or muscle group that is in close proximity.

**Purpose:**

The aim of the present study was two-fold: i) to quantify the level of crosstalk in the mechanomyographic (MMG) signals from the longitudinal (L_o_), lateral (L_a_) and transverse (T_r_) axes of the extensor digitorum (ED), extensor carpi ulnaris (ECU) and flexor carpi ulnaris (FCU) muscles during isometric wrist flexion (WF) and extension (WE), radial (RD) and ulnar (UD) deviations; and ii) to analyze whether the three-directional MMG signals influence the level of crosstalk between the muscle groups during these wrist postures.

**Methods:**

Twenty, healthy right-handed men (mean ± SD: age = 26.7±3.83 y; height = 174.47±6.3 cm; mass = 72.79±14.36 kg) participated in this study. During each wrist posture, the MMG signals propagated through the axes of the muscles were detected using three separate tri-axial accelerometers. The *x*-axis, *y*-axis, and *z*-axis of the sensor were placed in the L_o_, L_a_, and T_r_ directions with respect to muscle fibers. The peak cross-correlations were used to quantify the proportion of crosstalk between the different muscle groups.

**Results:**

The average level of crosstalk in the MMG signals generated by the muscle groups ranged from: 34.28–69.69% for the L_o_ axis, 27.32–52.55% for the L_a_ axis and 11.38–25.55% for the T_r_ axis for all participants and their wrist postures. The T_r_ axes between the muscle groups showed significantly smaller crosstalk values for all wrist postures [*F* (2, 38) = 14–63, p<0.05, *η* ^2^ = 0.416–0.769].

**Significance:**

The results may be applied in the field of human movement research, especially for the examination of muscle mechanics during various types of the wrist postures.

## Introduction

The mechanomyography (MMG) technique has not only been employed as an alternative non-invasive tool to surface electromyography (sEMG) recently [1], [2], but also provides additional information on motor unit recruitment and its firing rate for evaluating the conditions of muscle function [3], [4]. However, some factors limit using the MMG technique for a comprehensive examination of muscle activity [5], [6]. For example, the crosstalk that occurs between adjacent muscles is one of the more important concerns associated with both MMG [5], [6] and sEMG techniques [7], [8]. In the field of EMG and MMG, crosstalk refers to the contamination of the signal from the muscle of interest by the signal from another muscle or muscle group in close proximity [9].

When measuring the extent of crosstalk between the MMG signals of different muscles, the most interesting issue is the propagation direction of the mechanical waves that form the MMG signal.

Very little is known to date on exactly how muscle waves propagate. It is thought that mechanical waves travel in all directions away from the source (in the case of muscle, the fibers are the source of the wave generation) and are filtered by the surrounding objects (i.e., skin, adipose tissues, fascia, tendon, and bone) [10]. In contrast, researchers have shown that the MMG signal reflects low-frequency lateral (L_a_) oscillations of active skeletal muscle fibers [11]–[13]. Cescon *et al.* found that an MMG signal generated by a single motor unit propagates in the transverse (T_r_) direction, but not in the longitudinal (L_o_) direction, at the location of the sensor with respect to the muscle fibers [14]. Another study found that the MMG signal propagates both the L_o_ and T_r_ to the muscle fiber direction [15]. These authors [15] also found that the MMG amplitude decreases as it travels in the T_r_ direction. Archer *et al.* [16] found that MMG signals mainly propagate along the L_o_ direction of the muscle fibers if the frequencies are greater than 25 Hz and mainly in the T_r_ direction if the frequencies are less than 25 Hz. Interestingly, Farina *et al.* reported that single motor units generate heterogeneous surface MMG signals over the skin surface [17]. The authors also found that the peak-to-peak amplitude of the MMG signal depended on the motor unit location and the T_r_ axis of the accelerometer. However, the mean frequency of the MMG signal was dependent on both the T_r_ and L_o_ axes of the sensor with respect to muscle fibers [17].

Taken together, these reports provide a foundation of data for analyzing the crosstalk between MMG signals detected from multiple directions. Although some studies [6], [18] have examined the crosstalk of MMG signals from quadriceps, those studies did not examine the propagation axes of the muscle. This is important because crosstalk is highly associated with the propagation properties of muscle fiber oscillation and becomes even more critical for muscles that in close proximity with each other [6], [10]. For instance, the human forearm consists of several muscles in close proximity, and thus there is a relatively small surface area on the forearm for placing the recording devices. Additionally, the physiological interpretation of the signal generated by the forearm muscle of interest is difficult [7], [19].

To date, no previous study has examined crosstalk between the MMG signals detected from the forearm muscles. A limited number of studies have investigated the crosstalk in EMG signals from the forearm muscles [7], [20], [21], but none of them considered the propagation properties of the muscle fibers. Therefore, the present study poses two interrelated research questions:

- Does crosstalk occur for MMG signals generated by the forearm muscles from different axes during all wrist postures?
- Do any of the multi-axis MMG signals alter the level of crosstalk over a range of wrist postures?

Our hypothesis suggests that the multi-axis MMG signals from forearm muscles may show different levels of crosstalk due to the effects of the oscillation properties of muscle fibers forming the MMG signal. Additionally, any of these axes may accumulate less crosstalk, because the contaminated signals coming from the different directions of the adjacent muscles or muscle groups should not be the same in size.

One marked challenge in any discussion of crosstalk is how to measure or quantify it. Although a cross-correlation function has been criticized in previous studies [22], [23], it is now the most powerful method for quantifying crosstalk [6], [24]. Interestingly, both amplitude- and correlation-based indices were not statistically significant for the purpose of crosstalk quantification [23]. Nevertheless, the peak correlation coefficients (*R_x_*_, y_) at zero-phase shift are used as a cross-correlation function to quantify crosstalk because it is easier to use and can measure the proportion of a common signal between two muscles without knowing any information regarding an uncontaminated signal [7], [24]. The percentage crosstalk (*R^2^_x, y_*), which is the shared variance or percentage of common signal between two signals of adjacent muscle, may be computed by squaring the peak correlation to determine the common signal (% crosstalk) components between the two muscles [21], [24]. Therefore, the aim of the present study was twofold: i) to quantify the level of crosstalk in the MMG signals generated by the L_o_, L_a_, and T_r_ axes over the extensor digitorum (ED), extensor carpi ulnaris (ECU), and flexor carpi ulnaris (FCU) muscles during wrist flexion (WF) and extension (WE), as well as radial (RD) and ulnar (UD) deviations, and ii) to analyze whether the three-directional MMG signals alter the level of crosstalk between the muscle groups during all wrist postures.

## Methods

### Participants

Twenty, randomly selected right-handed healthy male volunteers (mean ± SD: age = 26.7±3.83 y; height = 174.47±6.3 cm; mass = 72.79±14.36 kg) provided written consent prior to their participation in this study after being fully informed of the purpose of the investigation and the experimental protocols. All of the participants were clinically healthy with no previous or ongoing records of neuromuscular or skeletomuscular disorders specific to the elbow, wrist, or finger joints.

### Ethics

This study was approved (Ref No.: KKM/NIHSEP/P13-685) by the local Medical Research & Ethics Committee (MREC), Ministry of Health, Bangsar, Kuala Lumpur, Malaysia, and was performed in accordance with the principles of the Declaration of Helsinki.

### Muscle contraction protocols

**Figure 1:**
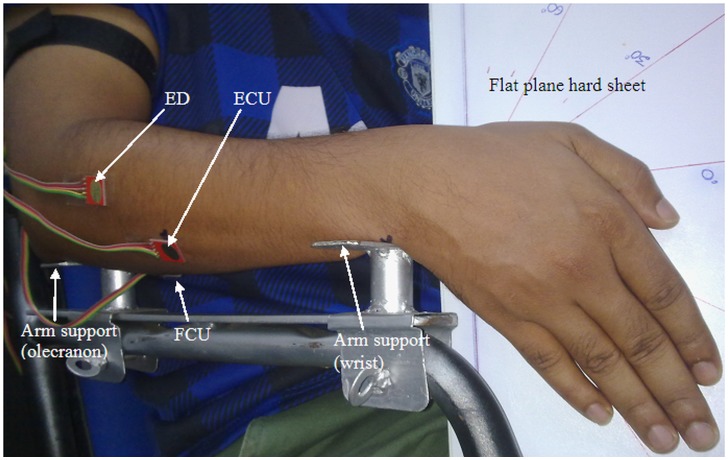
Schematic of the sensor placement to quantify the crosstalk in the MMG signals from the three axes of the ED, ECU, and FCU muscles during the UD wrist posture.

During the experiment, the participants were seated comfortably on a chair with two adjustable arm supports attached to the chair arm. Each participant's forearm was placed on the arm supports with a neutral posture. The ulna bone positioned near the wrist and elbow (olecranon) was used to fix the arm supports to ensure no contact pressure was made between the forearm muscles and the chair arm and to ensure comfortable wrist postures. The participants were required to execute three trials of the maximum angular range of WF and WE as well as RD and UD postures for 6 s, each with 2 min of rest between each set of contractions and trials. Of these three trials, the maximal angular range posture was used for each wrist posture. A standard finger posture between participants was used for each wrist posture (Fig. 1). A flat plain hard sheet with angular sketch, which was adjustable with each wrist posture, was used to determine angles of the wrist posture and to mitigate any off-axis wrist postures by participants (Fig. 1). The neutral position of the forearm was chosen because the maximal wrist range of motion occurs near this posture [25]. All of the wrist postures were performed at a joint angle of approximately 90° between the arm and the forearm and all angles were measured using an analog goniometer.

### MMG measurements

**Figure 2:**
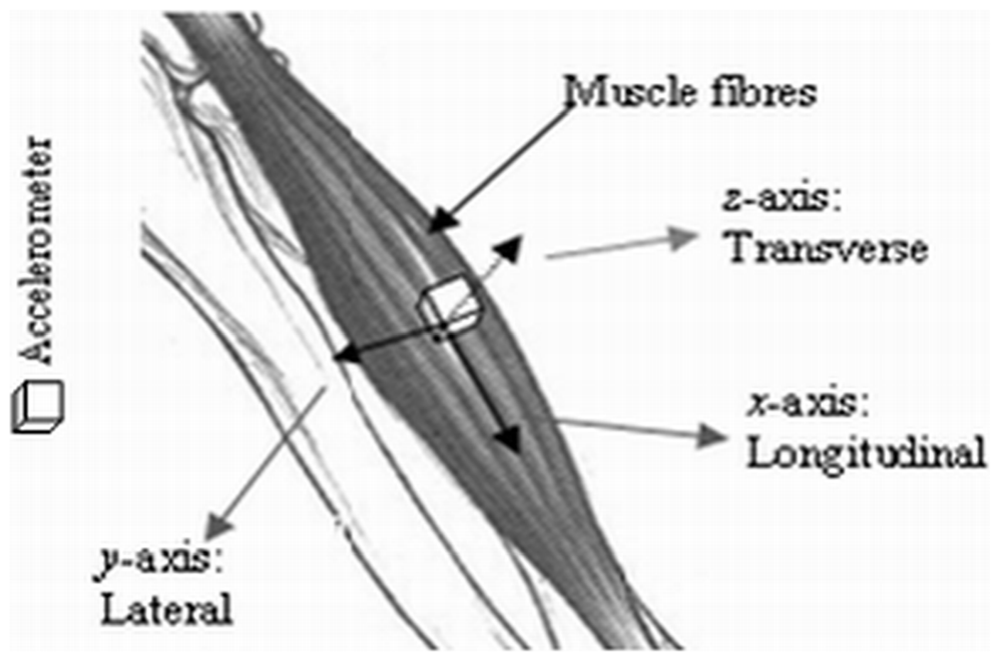
Schematic of the orientations of the L_o_, L_a_, and T_r_ axes of sensor with respect to muscle fiber to detect the MMG signals.

Three separate tri-axial accelerometers (ADXL335, Analog Devices, USA; full-scale range = ±3 g; typical frequency response = 0.5–500 Hz; sensitivity = 330 mV/g; size = 15 mm×15 mm×1.5 mm, including the breakout board on which it was mounted; weight including wires and breakout board <1.5 g) were used to detect the MMG signals. The accelerometers were attached to the skin surface over the muscle bellies of the ED, ECU, and FCU with double-sided adhesive tape during the neutral posture (Fig. 1). The *x*-axis, *y*-axis, and *z*-axis of each accelerometer were positioned in the L_o_, L_a_ and T_r_ directions to the muscle fibers, respectively (Fig. 2). The anatomical position of each muscle belly was determined according to the anatomical guide for the electromyographer by Perotto and Delagi, 2005 [26] as follows: ED – one third of the distance from the proximal end of a line from lateral epicondyle of the humerus to the distal head of the ulna; ECU – just lateral to the ulnar border that is half of the distance between the lateral epicondyle of the humerus and distal head of the ulna; and FCU – two finger widths from the ulnar border that is one third of the distance between the medial epicondyle of the humerus and distal head of the ulna (Fig. 1).

### Data acquisition and signal processing

The outputs of each direction of the three sensors were connected to the data acquisition unit (NI cDAQ 9191 wireless device and NI 9205 module with 16-bit resolution at CMMR of 100 dB; National Instruments, Austin, TX, USA), which differentially recorded the raw data at a rate of 1000 samples/s and stored the data in a computer for subsequent analyses. The raw data detected by the sensors from the L_o_, L_a_ and T_r_ directions with the muscle fibers were digitally bandpass-filtered (fourth-order Butterworth) at 5–100 Hz to obtain the MMG signals. The MMG signals were extracted for a 2-s period corresponding to the middle 33% of each 6-s wrist posture. Only the middle portions of data were analyzed to avoid signal of the transition period, which was when the muscle acted from rest to activity and vice versa as previously recommended [6], [27]. The 2-s segments for each MMG signal generated by the three axes of the muscles were used to quantify the crosstalk using the cross-correlation function. The cross-correlation values of the two signals *X_t_* and *Y_t_* in three directions were determined according to the following equation:
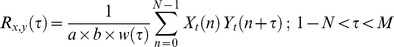
(1)where 
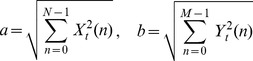
, *w* is the weighting factor, *M* and *N* are the lengths of *X_t_* and *Y_t_*, respectively, and *τ* represents the time lag between the signals.The peak cross-correlation coefficients were squared to obtain the magnitude of the crosstalk, *R* ^2^ *_x, y_* (i.e., common signal %) in MMG signals between the same general axes (e.g., between the L_o_ axes of two muscles) of the muscle groups that were investigated. All of the signal processing was performed with custom programs written in the LabVIEW programming software (version 12.0, National instruments, Austin, TX, USA).

### Data analysis

One-Way Repeated Measures Analysis of Variance (ANOVA) followed by the Least Significant Difference (LSD) post-hoc tests were used among the crosstalk values in the MMG signals from the three axes of the muscle groups during different wrist postures. The statistical analyses were performed using SPSS software (IBM SPSS Statistics, version 20, New York, USA). The critical value of F-ratio statistics, *F*_c_ = 3.25 at a significance level of α = 0.05, was affixed for statistical significant analysis. Therefore, a *p*≤0.05 was considered statistically significant.

## Results

**Figure 3:**
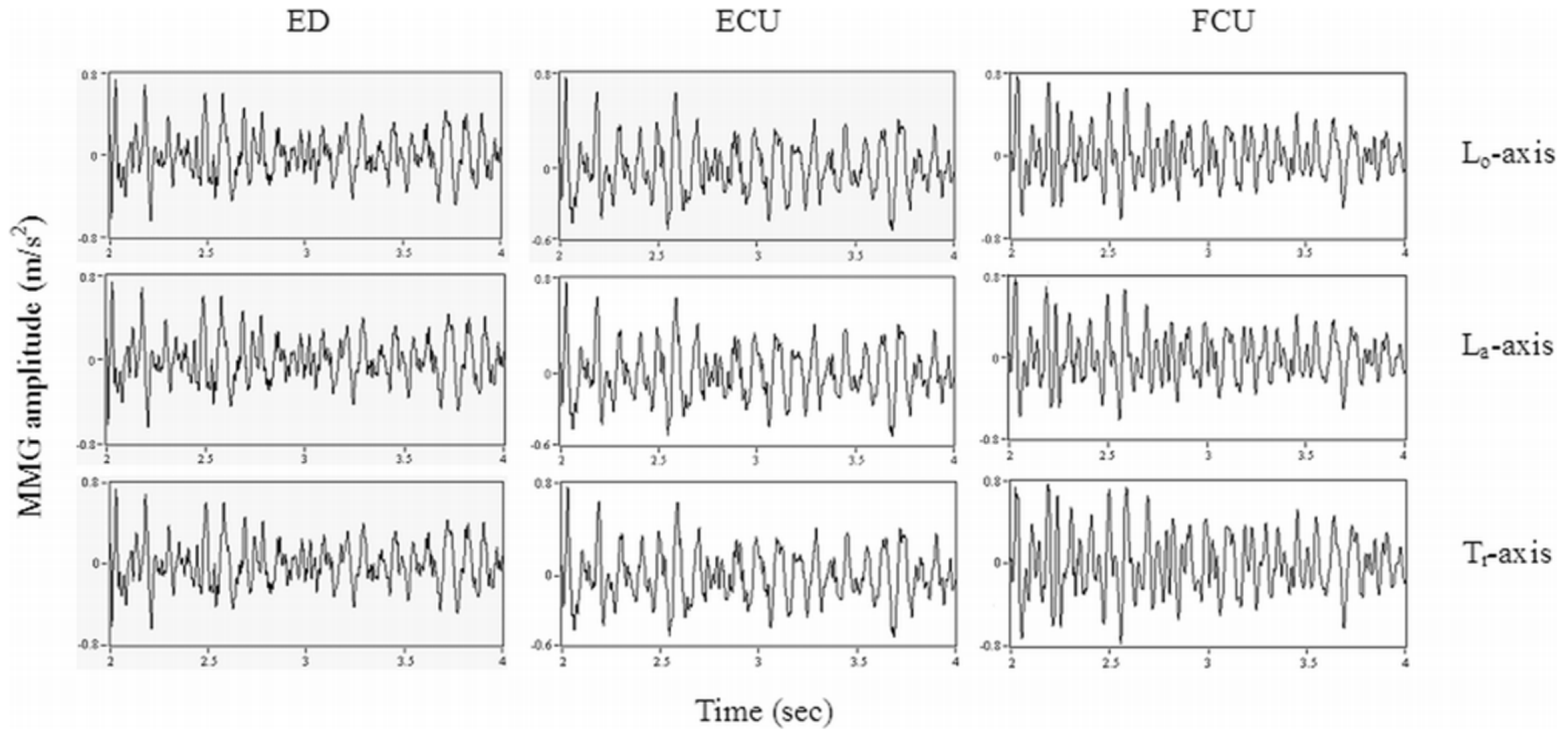
An example of the MMG signals from the three axes of the ED, ECU, and FCU muscles during the WE posture, which were used to quantify the crosstalk.

**Figure 4:**
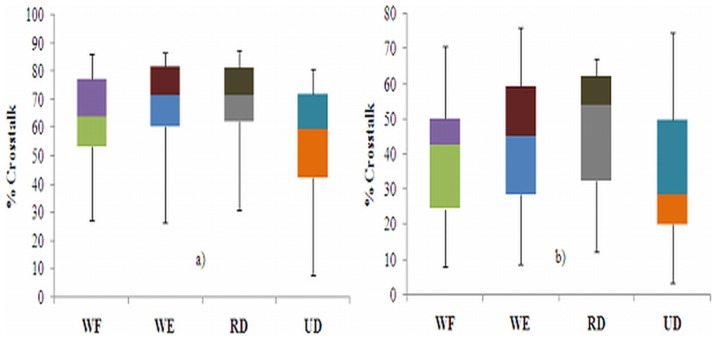
Box whisker plots of crosstalk in the MMG signals generated by each L_o_-axis between the a) ED and ECU as well as b) ECU and FCU muscle groups during the wrist postures.

**Figure 5:**
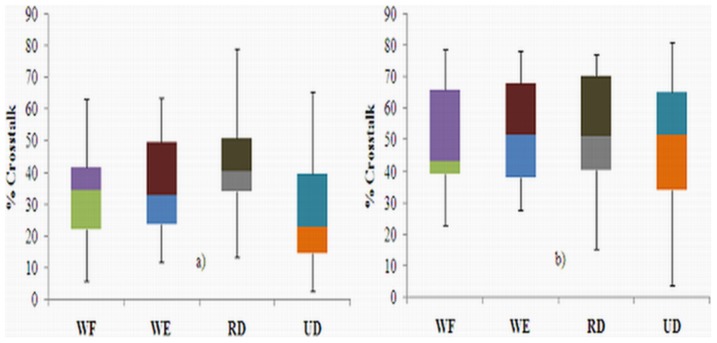
Box whisker plots of crosstalk in the MMG signals generated by each L_a_-axis between the a) ED and ECU as well as b) ECU and FCU muscle groups during the wrist postures.

**Figure 6:**
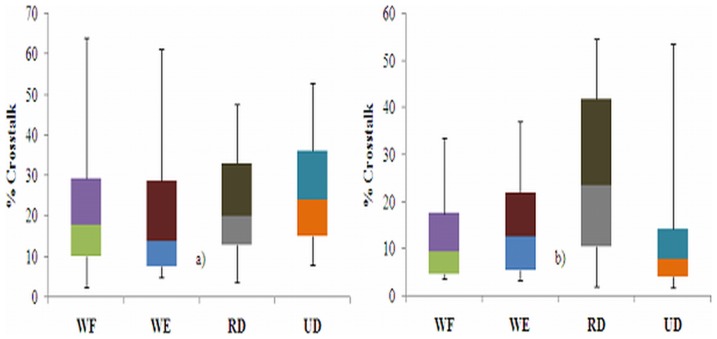
Box whisker plots of crosstalk in the MMG signals generated by each T_r_-axis between the a) ED and ECU as well as b) ECU and FCU muscle groups during the wrist postures.

Figure 3 shows an example of the MMG signals from the L_o_, L_a_, and T_r_ axes of the ED, ECU, and FCU muscles during the WE for one participant, which were used to quantify the crosstalk. Figures 4–6 show the box whisker plots for crosstalk values among the three directions of the MMG signals between the ED and ECU as well as the ECU and FCU muscle groups, which were obtained for each participant and each wrist posture. The mean level of crosstalk in the MMG signals generated by the three axes of the muscle groups ranged from *R* ^2^ *_x, y_* = 34.28–69.69% for the L_o_ axis, 27.32–52.55% for the L_a_ axis, and 11.38–25.55% for the T_r_ axis for all participants and wrist postures (Figs. 4–6). In all of the cross-correlation analyses, almost all of the peak coefficients appeared at a time shift of approximately 0 s (i.e., τ = 0 s).

**Figure 7:**
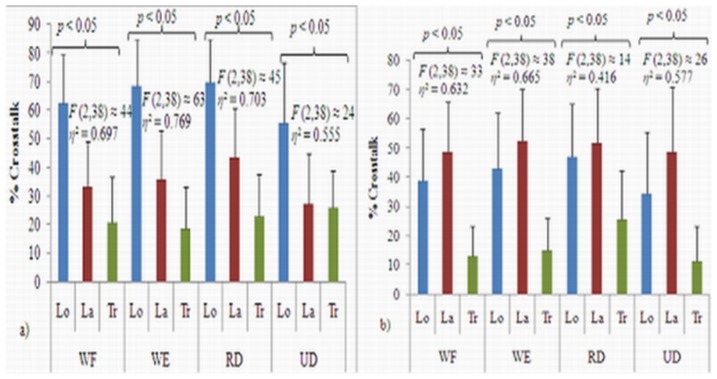
Schematics of One-Way Repeated Measures ANOVA analyses for the crosstalk values among the L_o_, L_a_, and T_r_ axes between the a) ED and ECU as well as b) ECU and FCU muscle groups during each wrist posture.

Figure 7 shows statistical analyses of the crosstalk values of the L_o_, L_a_, and T_r_ axes for the muscle groups during each wrist posture. These findings suggest that the T_r_ axis MMG signals for all of the muscle groups showed significantly lower crosstalk values for each wrist posture (*p*<0.05). The L_o_ axis between the ED and ECU muscles showed significantly greater crosstalk (*p*<0.05), whereas the L_a_ axis between the ECU and FCU muscles displayed significantly greater crosstalk values for all of the wrist postures (*p*<0.05). According to Cohen's interpretation of effect size for *F*-ratio statistics, when *p*≤0.05, Eta squared (*η* ^2^) is used to determine effect size as follows: 0.01 = small, 0.06 = medium and 0.138 = large [28], the crosstalk values between the MMG signals for the three axes showed large effect sizes (*η* ^2^ range = 0.30–0.81) for all of the wrist postures (Fig. 7). In addition, post-hoc analyses shown in Table 1 further confirmed that the crosstalk values in the MMG signals between the T_r_ axes of the muscle groups were significantly lowered for almost all of the wrist postures that were performed (*p*<0.05).

**Table 1:** LSD post-hoc analyses of the crosstalk values among the L_o_, L_a_, and T_r_ axes of the muscles during each wrist posture.

Wrist postures	|Mean crosstalk difference between axes|			Std. Error		p-value	
WF	L_o_-L_a_	28.95*	9.85#	4.991*	5.123#	0.000*	0.07#
	L_a_-T_r_	12.5	35.75	4.411	3.896	0.011	0.000
	T_r_-L_o_	41.45	25.9	4.202	4.6	0.000	0.000
WE	L_o_-L_a_	32.7	9.54	4.556	5.02	0.000	0.073
	L_a_-T_r_	17.4	37.53	5.101	4.6	0.003	0.000
	T_r_-L_o_	50.3	27.99	3.813	3.766	0.000	0.000
RD	L_o_-L_a_	26.2	4.6	5.12	5.32	0.000	0.4
	L_a_-T_r_	20.44	26.2	5.183	6.03	0.001	0.000
	T_r_-L_o_	46.64	21.57	4.45	4.662	0.000	0.000
UD	L_o_-L_a_	28.3	14.40	4.62	4.07	0.000	0.002
	L_a_-T_r_	1.4	37.32	4.505	5.92	0.754	0.000
	T_r_-L_o_	29.7	22.91	5.492	5.514	0.000	0.001

The columns followed by ‘#’ present the data between the ECU and FCU muscles.

The columns followed by ‘*’ present the data between the ED and ECU muscles.

## Discussion

The present study quantified the level of crosstalk between the MMG signals generated by the L_o_, L_a_, and T_r_ axes of the ED, ECU, and FCU muscles during WF, WE, RD and UD wrist postures. Theoretically, the MMG signals from two muscles should not have a high level of crosstalk because there is a low possibility that a common signal from different muscles will have the same waveform shape [6]. Interestingly, the present study revealed that the level of crosstalk in the MMG signals detected from the three axes over the ED, ECU, and FCU muscle groups ranged from *R* ^2^ *_x, y_* = 11.38% to 69.69% (Figs. 4–6).This assessment is in agreement with the findings of Mogk and Keir [20] who showed that the crosstalk in sEMG signals generated by forearm muscles reached 58% for flexors and 64% for extensors during gripping tasks. In addition, the magnitude of the crosstalk obtained in this study can be compared to the findings reported by Beck *et al.* [6] who found that the peak correlation coefficients between the superficial quadriceps femoris muscles ranged from 0.124 to 0.714 (i.e., 1.5 to 51% common signal) during sub-maximal to maximal isometric contractions. This difference in magnitude of the crosstalk may be because forearm muscles are relatively small compared to quadriceps femoris muscles and thus are in closer proximity, which would be expected to generate greater crosstalk. The present study supports the existence of crosstalk in the forearm and also suggests that the complete differentiation of activity from individual muscles is difficult in the forearm [19]. The level of crosstalk that we detected between the MMG signals generated by the forearm muscles may be because more than ten individual muscles act to flex and extend the phalanges and hand (i.e., extensor carpi radialis longus, extensor carpi radialis brevis, extensor carpi ulnaris, extensor pollicis brevis, flexor carpi ulnaris, and flexor pollicis longus). Together, these muscles may contribute to the MMG signals due to their close proximity and the small area to which the sensors were placed.

We also found that the level of crosstalk in the MMG signals among the three axes of the ED, ECU, and FCU muscles was significantly different across all of the wrist postures (*p*<0.05).The differences in crosstalk values among the MMG signals from the three axes showed large effect sizes for wrist postures (Fig. 7). Hence, there is strong statistical evidence that the level of crosstalk differed significantly among all three axes for each muscle group and wrist posture. In addition, post-hoc analyses (Table 1) further confirm that the T_r_ axis MMG signals for the different muscle groups showed a significantly lower crosstalk values for almost all of the experimental conditions. Since the amplitude of MMG signal generated by the T_r_ axis of the muscle fibers attenuates the signal strength during travel through the direction. This may be the reason for the reduction in the level of crosstalk in that direction [15]. Thus, these results confirm our hypothesis that any of the three axes would accumulate lower crosstalk values because the contaminated signals coming from the different directions of the adjacent muscles or muscle groups should not be the same magnitude. In addition, the propagation components of the MMG signals along the three axes of those muscles may not be the same size or shape. It is possible that the MMG signals generated by forearm muscles may be influenced by the agonist and antagonist muscles as a result of wrist postures [29].

Our results have practical impact because the MMG technique has been widely used to examine motor control of prosthetic devices [30]–[32] and muscle mechanics during various types of muscle postures [1], [33]. These results may be useful for studies that use multi-axis MMG to examine muscle function [30], [34]–[36]. For example, Scheeren *et al.* [30] used MMG signals from three axes of the forearm to identify the WF, WE, RD, and UD wrist movements. The authors [30] reported that MMG signals generated by the forearm muscles can be used for motor prosthetic control during wrist movements. Hence, the results of this study may be used in the clinic for designing an MMG-driven prosthetic device. They may also be useful for monitoring the conditions of muscle function by increasing our understanding of forearm muscle mechanics during various wrist postures. Interestingly, our previous study demonstrated that wrist postures do not influence the level of crosstalk in the MMG signals generated by forearm muscles (unpublished data). Another study from our laboratory showed that the level of crosstalk in the MMG signals generated by the forearm muscles increases linearly as the degree of gripping force increases [37]. Importantly, the muscle groups studied in our previous work were the same as those presented here.Our results also provide a foundation for future research directions. First, it would be interesting to investigate whether the T_r_ axis MMG signals exhibit a lower level of crosstalk for a large number of forearm muscles. Second, future research should investigate whether theT_r_ axis MMG signal may be used to improve identification accuracy of wrist postures based on the crosstalk that the present study quantified. Additional research is needed to determine the effects of crosstalk on the MMG signals for examining the condition of both normal and abnormal muscle functions for different muscle groups using various types of static and dynamic muscle actions.

The present study has several possible limitations. First, the cross-correlation function used in this study cannot differentiate between the individual contributions of motor unit synchrony and the components of contaminated signals of two muscles [20]. Therefore, cross-correlations can represent crosstalk, but they can also represent other mechanisms. Second, the present study did not consider skin-fold thickness differences of the forearm, which cannot be ruled out as contributors since tissue thickness influences the MMG signal. This is a very important issue when discussing crosstalk and the propagation of mechanical waves through inactive tissues (e.g., subcutaneous fat, skin, and bone) [10].Therefore, as the level of crosstalk depends on the tissue thickness of the muscle of interest, the results of the present study may not hold true for other muscle groups.

## Conclusions

In summary, we conclude that the level of crosstalk in the MMG signals depends on the muscle fiber axes during wrist postures. The T_r_ axis MMG signals between the muscle groups showed significantly less crosstalk for each wrist posture. Therefore, the muscle axis needs to be accounted for when accurate identification of an individual muscle's activity in the forearm is of interest. Thus, these results may be useful in rehabilitative settings for assessing muscle activity, especially when studying motor control mechanisms, as well as in clinics for designing an MMG-driven prosthetic device.
